# Prediction of delayed graft function by early salivary microbiota following kidney transplantation

**DOI:** 10.1007/s00253-024-13236-w

**Published:** 2024-07-01

**Authors:** Xuyu Xiang, Bo Peng, Kai Liu, Tianyin Wang, Peng Ding, Yi Zhu, Ke Cheng, Yingzi Ming

**Affiliations:** 1 The Transplantation Center of the Third Xiangya Hospital, Central South University, Changsha, 410013 China; 2 Engineering and Technology Research Center for Transplantation Medicine of National Health Commission, Changsha, China

**Keywords:** Salivary microbiota, Delayed graft function, Early-stage prediction, Kidney transplantation, Perioperative period

## Abstract

**Abstract:**

Delayed graft function (DGF) is a frequently observed complication following kidney transplantation (KT). Our prior research revealed dynamic shifts in salivary microbiota post-KT with immediate graft function (IGF), yet its behavior during DGF remains unexplored. Five recipients with DGF and 35 recipients with IGF were enrolled. Saliva samples were collected during the perioperative period, and 16S rRNA gene sequencing was performed. The salivary microbiota of IGFs changed significantly and gradually stabilized with the recovery of renal function. The salivary microbiota composition of DGFs was significantly different from that of IGFs, although the trend of variation appeared to be similar to that of IGFs. Salivary microbiota that differed significantly between patients with DGF and IGF at 1 day after transplantation were able to accurately distinguish the two groups in the randomForest algorithm (accuracy = 0.8333, sensitivity = 0.7778, specificity = 1, and area under curve = 0.85), with *Selenomonas* playing an important role. *Bacteroidales* (Spearman’s *r* =  − 0.4872 and *p* = 0.0293) and *Veillonella* (Spearmen’s *r* =  − 0.5474 and *p* = 0.0125) were significantly associated with the serum creatinine in DGF patients. Moreover, the significant differences in overall salivary microbiota structure between DGF and IGF patients disappeared upon long-term follow-up. This is the first study to investigate the dynamic changes in salivary microbiota in DGFs. Our findings suggested that salivary microbiota was able to predict DGF in the early stages after kidney transplantation, which might help the perioperative clinical management and early-stage intervention of kidney transplant recipients.

**Key points:**

*• Salivary microbiota on the first day after KT could predict DGF.*

*• Alterations in salivary taxa after KT are related to recovery of renal function.*

**Supplementary Information:**

The online version contains supplementary material available at 10.1007/s00253-024-13236-w.

## Introduction

The end-stage renal disease (ESRD) is a significant public health issue caused by aging populations and a rise in chronic noncommunicable diseases. It results in high mortality rates and economic costs. Kidney transplantation (KT) is an effective treatment option that offers patients the potential for a return to a normal life. The waiting list for KT is steadily increasing, leading to a greater demand for deceased donor donations. Since the national implementation of civilian deceased organ donation on February 25, 2014, donation after a citizen’s death (DCD) became the sole source for an organ transplant in China (Huang et al. 2015). However, DCD is associated with a higher incidence of delayed graft function (DGF) (Bellini et al. 2022).

DGF is a frequently observed complication following DCD KT, and it is linked to inferior short-term and long-term outcomes, as well as high rejection rates (Nicholson et al. 1996; Geddes et al. 2002). DGF usually results in significant systemic complications that necessitate prompt diagnosis and intervention to prevent graft failure (Palmisano et al. 2021). Nonetheless, diagnostic criteria for DGF require a minimum of 72 h to establish a definitive diagnosis (Mallon et al. 2013). Thus, early-stage prediction plays a crucial role in improving the prognosis of patients suffering from DGF. Despite the existing indicators, such as urine volume, renal function, and resistance index of Doppler ultrasound, there remains a need for a non-invasive index that is accurate and sensitive to early evaluate the prognosis of patients with KT (Quaglia et al. 2020). There is a critical need for biomarkers that can aid in the early-stage prediction of DGF following kidney transplantation and facilitate the determination of patient prognosis to improve the management of complications and prolong graft survival.

As the pioneer center in exploring the clinical utility of salivary microbiota following KT, we have previously detected noteworthy alterations in salivary microbiota in patients with immediate graft function (IGF) during the early post-transplant phase. Moreover, we have identified *Patescibacteria* and *Leptotrichiaceae* that were associated with renal function (Xiang et al. 2023). Nevertheless, it remains unclear how the salivary microbiota of DGF patients varies, how it differs from that of IGF patients, and whether the salivary microbiota data of DGF patients possess clinical significance in prediction. While no specific research has yet directly explored the potential connection between the microbiota in the digestive tract and DGF, several studies have identified changes in salivary microbiota accompanying various kidney diseases (Ramanathan et al. 2023). Furthermore, salivary microbiota has been implicated in the occurrence and progression of various systemic diseases, such as diabetes, cancer (Belstrøm 2020), hypertension (Chen et al. 2023), Alzheimer’s disease (Lu et al. 2022), and serves as a non-invasive biomarker in their diagnosis (Zhang et al. 2016). These scientific findings further motivate our investigation into the role of salivary microbiota in DGF.

In the present study, we validated our previous findings using a larger dataset first. We investigated the salivary microbiota dynamic variation in patients with DGF and compared them with those of patients with IGF. Our findings suggested the potential of salivary microbiota as a biomarker for the early-stage (24-h) prediction of DGF and revealed the correlation between certain species and the recovery of DGF patients during the perioperative period.

## Materials and methods

### Study design

This was a retrospective, case–control study to evaluate the prediction of DGF with the detection of early-stage salivary microbiota. Both inpatients and outpatients underwent kidney transplantation from October 1, 2022, to April 1, 2023, in the Transplantation Center, The Third Xiangya Hospital, Central South University. The exclusion criteria included (1) age less than 18 years old or more than 65 years old, (2) multiple-organ transplantation, and (3) living-related kidney transplantation. All patients who were not excluded were enrolled. The study protocol was approved by the Ethics Committee of the Third Xiangya Hospital of Central South University, Changsha, China (No. 22207). Written informed consent was obtained from all study participants. Experiments were carried out following the ethical guidelines set by the Declaration of Helsinki 1964 and its later amendments.

To further investigate changes in the salivary microbiota of DGF recipients after their recovery, we collected samples after long-term follow-up from the original cohort, from March 4 to March 17, 2024. For both DGF and IGF recipients, inpatients were required to have serum creatinine levels below 176 µmol/L. However, due to factors such as graft failure and loss of follow-up, two DGF patients from the original cohort were excluded. Consequently, we expanded the recruitment criteria for DGF patients, including those who (1) underwent kidney transplantation in our center between 6 months and 2 years ago, (2) aged between 18 and 65 years, (3) did not receive kidney transplants from relatives or multiple organ transplants, and (4) had serum creatinine levels recovering to below 176 µmol/L.

### Sample collection

Saliva samples were collected at various time points before and after surgery, including 1 day, 3 days, 7 days, and 14 days for all patients, and the week when serum creatinine (Scr) fell below 400 μmol/L only for patients with DGF.

Before collection, patients fasted for half an hour and rinsed their mouths. Patients spit the saliva into a sterile tube until it reached 2 ml. Saliva was stored at − 80 ℃ immediately after collection.

### 16S rRNA sequencing and data analysis

The details of 16S rRNA sequencing and data analysis were consistent with our previous study (Xiang et al. 2023). In brief, DNA extraction was performed using a Magnetic Soil and Stool DNA Kit (TIANGEN, Beijing, Shanghai). The V3-V4 hypervariable regions of bacterial 16S ribosomal genes were amplified using specific primers (341F: CCTAYGG-GRBGCASCAG, 806R: GGACTACNNGGGTATCTAAT) with barcodes. Quantitative PCR (qPCR) was used for quantification, followed by sequencing on the NovaSeq 6000 platform (Illumina, San Diego, CA, USA). Operational taxonomic units (OTUs) were clustered at 97% sequence similarity and annotated using the Silva 132 database (Quast et al. 2013). Alpha and beta diversity were analyzed using in-house Perl scripts. Principal component analysis (PCA) was conducted using QIIME software (Bolyen et al. 2019), and principal coordinate analysis (PCoA) utilized weighted unifrac distance. Analysis of similarities (ADONIS) based on Bray–Curtis dissimilarity was performed. Linear discriminant analysis effect size (LEfSe) was employed to identify biomarkers between groups, with an absolute linear discriminant analysis (LDA) score > 2 as the cutoff.

### Model building

The classification ability of the salivary microbiota for DGF was analyzed through randomForest algorithm by R package “randomForest” (4.7–1.1) (Liaw and Wiener 2002) and “caret” (6.0–94) (Kuhn 2008).

The data was divided into training (70%) and testing (30%) data sets, and we developed a random forest algorithm on the training data set for the outcome variables (DGF or IGF), and then the model’s performance was tested with the testing data set. All significant taxa from the LEfSe analysis were included. The logarithmic form or original form of relative abundance of these taxa was selected as the target variable. Next, we ranked the importance of each variable. Finally, receiver operating characteristic curves (ROC curves) and the area under the curve for each of our models by using the testing data set were assessed.

### Statistical analysis

All statistical analyses were performed using R (4.2.1, Posit Software, Boston, MA, USA), SPSS software 26 (IBM, Chicago, IL, USA), and Graphpad Prism 8 (Graphpad Software, San Diego, CA, USA). We utilized various statistical tests including the Wilcoxon rank-sum test, unpaired *t*-test, Mann–Whitney test, chi-square test, Fisher’s exact test, and repeated measures analysis of variance to assess inter-group differences. Spearman test and multiple linear regression analysis were employed to analyze the taxa-Scr correlation. Binary logistic regression was used for taxa classification. Statistical significance was set at *p* < 0.05.

## Results

### Study population

From October 1, 2022, to April 1, 2023, a total of 44 ESRD patients who received kidney transplantation from DCD in our center were enrolled. A total of five patients developed DGF after transplantation. DGF was defined as the need for dialysis within 7 days post-kidney transplantation or Scr over 400 μmol/L on the seventh day after transplantation(6). Saliva samples were collected at 1 day (*n* = 3), 3 days (*n* = 5), 7 days (*n* = 5), 14 days (n = 2) after surgery, and the week when Scr fell below 400 μmol/L (*n* = 5). The samples with Scr greater than 400 were classified into the DGF group, while the rest formed the DGF_Re group.

Since all DGF patients received antihuman thymocyte globulin (ATG) and tacrolimus, 35 patients with IGF were screened from the remaining 39 patients according to the same induction and immunosuppressive treatment regimen. Saliva samples were collected at 1 day (IGF_1D, *n* = 28), 3 days (IGF_3D, *n* = 28), 7 days (IGF_7D, *n* = 35), and 14 days (IGF_14D, *n* = 23) after surgery.

Moreover, a total of 27 saliva specimens were collected before kidney transplantation from these 40 patients, forming the ESRD group.

The clinical information of DGF (age 39.20 ± 4.49 years, 100% males) and IGF (age 43.34 ± 11.48 years, 71.43% males) groups is shown in Table 1. The mean body weight index (BMI) was 24.11 ± 4.26 kg/m^2^ for DGFs and 22.12 ± 3.78 kg/m^2^ for IGFs. All patients received ATG for induction, the same triple immunosuppressive therapy including tacrolimus, mycophenolic acid and steroids, and β-lactam antibiotics as primary antibiotics. There was no significant difference in each clinical data between the DGF and IGF groups except for Scr after kidney transplantation.

**Table 1:** General characteristics of DGFs and IGFs

	DGF (*n* = 5)	IGF (*n* = 35)	*p*
Gender, *n* (%)			0.1675^#^
Male	5 (100.00%)	25 (71.43%)	
Female	0	10 (28.57%)	
Age (years), mean ± SD	39.20 ± 4.49	43.34 ± 11.48	0.4788
Body mass index (kg/m^2^), mean ± SD	24.11 ± 4.26	22.12 ± 3.78	0.4328
Induction therapy, *n* (%)			
ATG	5 (100.00%)	35 (100.00%)	
Immunosuppressive therapy, *n* (%)			
Tacrolimus + mycophenolic acid + steroids	5 (100.00%)	35 (100.00%)	
Antibiotics, *n* (%)			0.4615
Beta-lactam	5 (100.00%)	35 (100.00%)	
Engpolymyxin	2 (40.00%)	5 (14.29%)	
Anti-fungal drugs	1 (20.00%)	10 (28.57%)	
Dialysis type, *n* (%)			0.6162^#^
HD	4 (80.00%)	21 (60.00%)	
PD	1 (20.00%)	10 (28.57%)	
None	0	4 (11.43%)	
Dialysis duration (months), mean ± SD	37.00 ± 29.52	18.61 ± 15.87	0.1505
Cause of end stage renal disease, n (%)			0.7932^#^
IgA nephropathy	0	2 (5.71%)	
Ssclerosing glomerulonephritis	0	1 (2.86%)	
Unknown	5 (100.00%)	32 (91.43%)	
Scr before KT (μmol/L), mean ± SD	1303.00 ± 265.80	1053.00 ± 344.70	0.1022
eGFR before KT (mL/min/1.73 m^2^), mean ± SD	3.83 ± 1.03	4.86 ± 2.08	0.4044
Scr after KT (μmol/L), mean ± SD			< 0.0001^*^
1 day	1431.00 ± 169.50	896.40 ± 348.80	
3 days	1250.00 ± 322.10	403.70 ± 379.30	
7 days	855.40 ± 562.30	167.70 ± 87.61	
14 days	613.60 ± 581.60	119.00 ± 37.63	

*ATG*, antihuman thymocyte globulin; *HD*, hemodialysis; *PD*, peritoneal dialysis; *Scr*, serum creatinine; *eGFR*, estimated glomerular filtration rate

^#^Analyzed with Fisher’s exact test; *analyzed with methods repetitive measure analysis of variance

All *p* values less than 0.05 were highlighted in bold

### The dynamic change of salivary microbiota in patients with IGF during the early-stage post-kidney transplantation

**Fig. 1:**
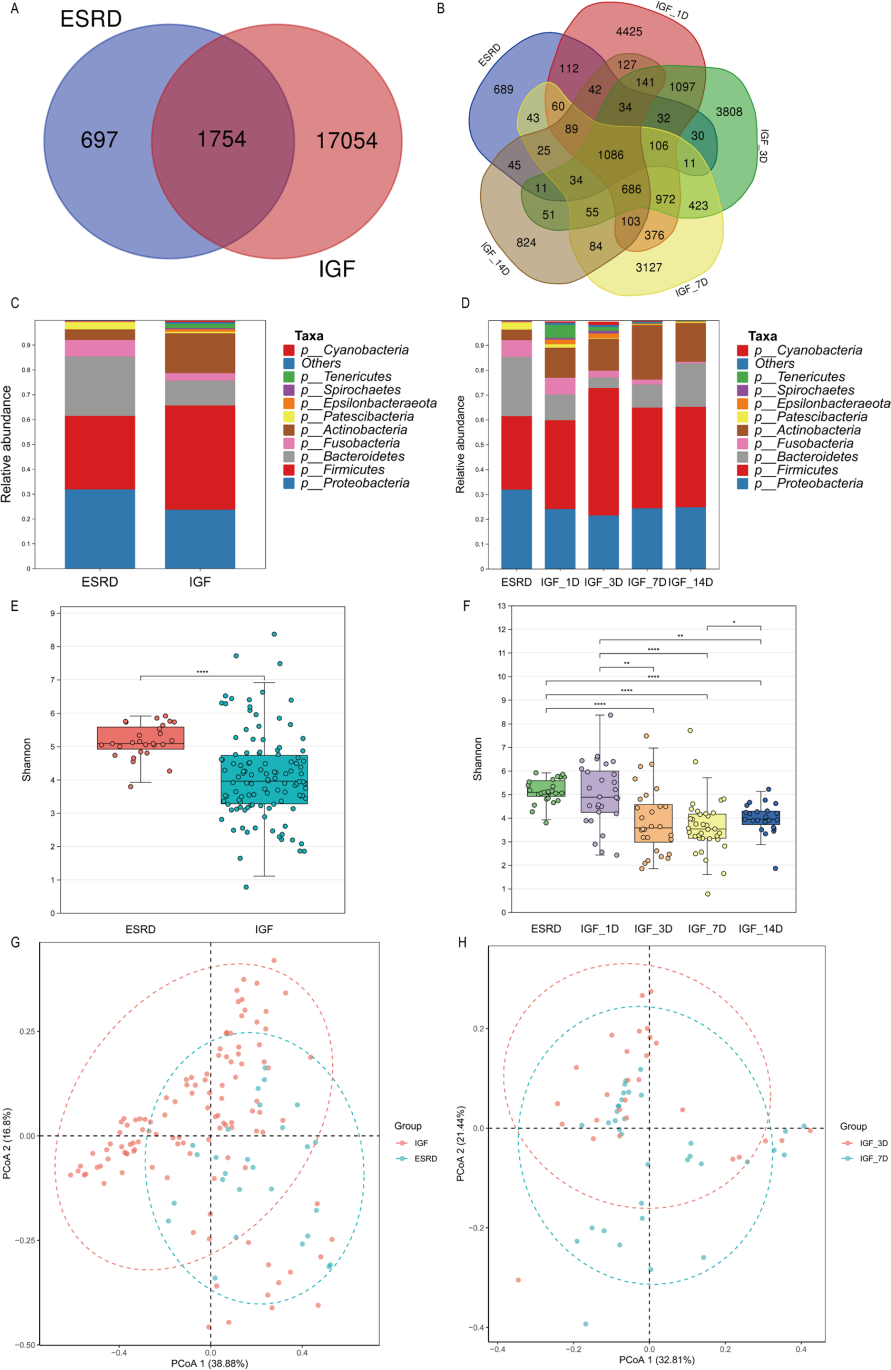
Composition characteristics of salivary microbiota in ESRDs and IGFs at different time points and states: **A** Venn graph for the group OTUs of ESRD and IGF groups; **B** Venn graph for the group OTUs of ESRD, IGF_1D, IGF_3D, IGF_7D, and IGF_14D groups; **C** salivary species composition of ESRD and IGF groups at phylum level; **D** salivary species composition of ESRD, IGF_1D, IGF_3D, IGF_7D, and IGF_14D groups at phylum level; **E** Shannon index of ESRD and IGF groups; **F** Shannon index of ESRD, IGF_1D, IGF_3D, IGF_7D, and IGF_14D groups; **G** PCoA graph of ESRD and IGF groups; **H** PCoA graph of IGF_3D and IGF_7D groups. ESRD, end-stage renal disease; IGF, immediate graft function; OTU, operational taxonomic unit; PCoA, principal coordinate analysis

Firstly, we validated the changes in salivary microbiota in IGF patients as in our previous research using a larger sample size (Xiang et al. 2023). Figure 1 A and B show the number of group OTUs. Our results indicated that the IGF patients had a higher number of group OTUs than ESRD patients, and the number of group OTUs decreased over time in the IGF group. The pairwise comparisons of the number of sample OTUs (Supplemental Fig. S1) were performed between these groups, and comparisons with a significant difference were as follows: IGF_1D vs. ESRD, *p* = 0.0062; IGF_7D vs. ESRD, *p* = 0.0088; IGF_14D vs. ESRD, *p* = 0.0034; IGF_1D vs. IGF_7D, *p* = 0.0001; IGF_1D vs. IGF_14D, *p* = 0.0001. At the phylum level, the composition of salivary microbiota in the IGF patients was quite different from that of the ESRD patients, and the microbial composition in the IGF group changed over time (Fig. 1 C and D). The Shannon index was used to reflect the richness and evenness of the microbiota. We observed a significant difference between the IGF and ESRD groups (*p* < 0.0001) and a greater within-group difference in the IGF patients (Fig. 1E). The postoperative time also affected the value of the Shannon index and the intra-group differences (Fig. 1F). The Shannon index of the ESRD group was significantly different from groups IGF_3D, IGF_7D, or IGF_14D, and all *p* values were less than 0.0001. There were also significant differences between IGF_1D vs. IGF_3D (*p* = 0.0023), IGF_1D vs. IGF_7D (*p* < 0.0001), and IGF_1D vs. IGF_14D (*p* = 0.001) and between IGF_7D vs. IGF_14D (*p* = 0.0351) but not between IGF_3D vs. IGF_7D (*p* = 0.6954) and IGF_3D vs. IGF_14D (*p* = 0.3718). As the postoperative time increased, the Shannon index and within-group differences became smaller. To assess of beta diversity of microbiota, we used weighted unifrac distance for PCoA and ADONIS to identify differences in microbial communities between two groups. There was a significant difference between the groups of ESRD vs. IGF (Fig. 1G, p = 0.001), ESRD vs. IGF_1D (*p* = 0.001), IGF_1D vs. IGF_3D (*p* = 0.008), and IGF_3D vs. IGF_7D (Fig. 1H, p = 0.002). No significant difference was shown between the groups IGF_7D and IGF_14D (*p* = 0.097). These findings are consistent with our previous study (Xiang et al. 2023).

We then conducted a correlation analysis to investigate the relationship between the abundance of each taxon and the Scr level. Spearman test was first performed for all taxa and Scr as the univariate analysis. A total of 584 taxa (28.40%) significantly correlated with Scr in all levels. Then taxa with the absolute values of Spearman’s *r* > 0.4 and *p* < 0.0001 were selected, and the multiple linear regression analysis was performed between these taxa and Scr levels (Table 2). We found that *Dialister*, *F0058*, *Parvimonas*, *Selenomonas*, and *Selenomonas* 4 were significantly and independently associated with Scr levels.

**Table 2:** The correlation between the salivary microbiota and Scr level in patients with IGF after kidney transplantation

	Univariate analysis		Multivariate analysis	
	Spearman’s *r*	*p*	Standard *β*	*p*
*p__Patescibacteria*	0.4853	** < 0.0001**	1.9430	0.0554
*c__Mollicutes*	0.5341	** < 0.0001**	0.8979	0.3719
*o__Campylobacterales*	0.5352	** < 0.0001**	0.4062	0.6856
*o__Spirochaetales*	0.4514	** < 0.0001**	1.1090	0.2708
*o__Synergistales*	0.4672	** < 0.0001**	0.7594	0.4498
*f__Atopobiaceae*	0.4570	** < 0.0001**	0.4933	0.6231
*f__FamilyXIII*	0.4030	** < 0.0001**	0.4358	0.6641
*f__Lachnospiraceae*	0.4467	** < 0.0001**	0.9369	0.3516
*f__Peptococcaceae*	0.4482	** < 0.0001**	0.1871	0.8520
*f__Propionibacteriaceae*	0.4151	** < 0.0001**	1.5260	0.1308
*f__Saccharimonadaceae*	0.5220	** < 0.0001**	1.6060	0.1120
*g__Campylobacter*	0.4998	** < 0.0001**	0.3727	0.7103
*g__Cardiobacterium*	0.4398	** < 0.0001**	1.0160	0.3128
*g__Clostridialesbacteriumcanineoraltaxon162*	0.4601	** < 0.0001**	1.1850	0.2396
*g__Comamonas*	0.4796	** < 0.0001**	0.5658	0.5731
*g__Corynebacterium*	0.4364	** < 0.0001**	0.0287	0.9771
*g__Desulfobulbus*	0.4149	** < 0.0001**	1.1140	0.2686
*g__Dialister*	0.5005	** < 0.0001**	2.9630	**0.0040**
*g__F0058*	0.5396	** < 0.0001**	2.4550	**0.0162**
*g__Fretibacterium*	0.4205	** < 0.0001**	0.2954	0.7684
*g__Johnsonella*	0.6204	** < 0.0001**	0.7936	0.4297
*g__Mogibacterium*	0.4951	** < 0.0001**	1.2070	0.2308
*g__Mycoplasma*	0.5115	** < 0.0001**	0.9140	0.3634
*g__Olsenella*	0.5391	** < 0.0001**	0.1809	0.8569
*g__Parvimonas*	0.5734	** < 0.0001**	5.0230	** < 0.0001**
*g__RuminococcaceaeUCG-014*	0.4528	** < 0.0001**	1.7900	0.0772
*g__Selenomonas*	0.5234	** < 0.0001**	3.5580	**0.0006**
*g__Selenomonas3*	0.5116	** < 0.0001**	1.0390	0.3019
*g__Selenomonas4*	0.5628	** < 0.0001**	4.5550	** < 0.0001**
*g__Tannerella*	0.4908	** < 0.0001**	1.3480	0.1814
*g__Treponema2*	0.5000	** < 0.0001**	0.5419	0.5893

*IGF*, immediate graft function

All *p* values less than 0.05 were highlighted in bold

### Comparison of salivary microbiota between DGF, ESRD, and IGF patients

The salivary microbiota at each time point from the DGF group was compared first, and the result is shown in Supplemental Fig. S2. It showed a minor difference in salivary microbiota composition between different time points. On the other hand, these specimens were collected from patients in a state of renal dysfunction (Scr > 400 μmol/L), and previous studies have shown a significant correlation between salivary microbiota composition and Scr in patients after kidney transplantation which implied that these samples share a similar colony distribution. Therefore, the timepoints were collapsed, and the samples of the DGF group were merged for analysis. This group included nine specimens from three patients on 1 day, 3 days, and 7 days after surgery and six specimens from two patients on 3 days, 7 days, and 14 days after surgery. Thus, the group was balanced to include three samples for each patient which helped weight the taxa of patients evenly.

**Fig. 2:**
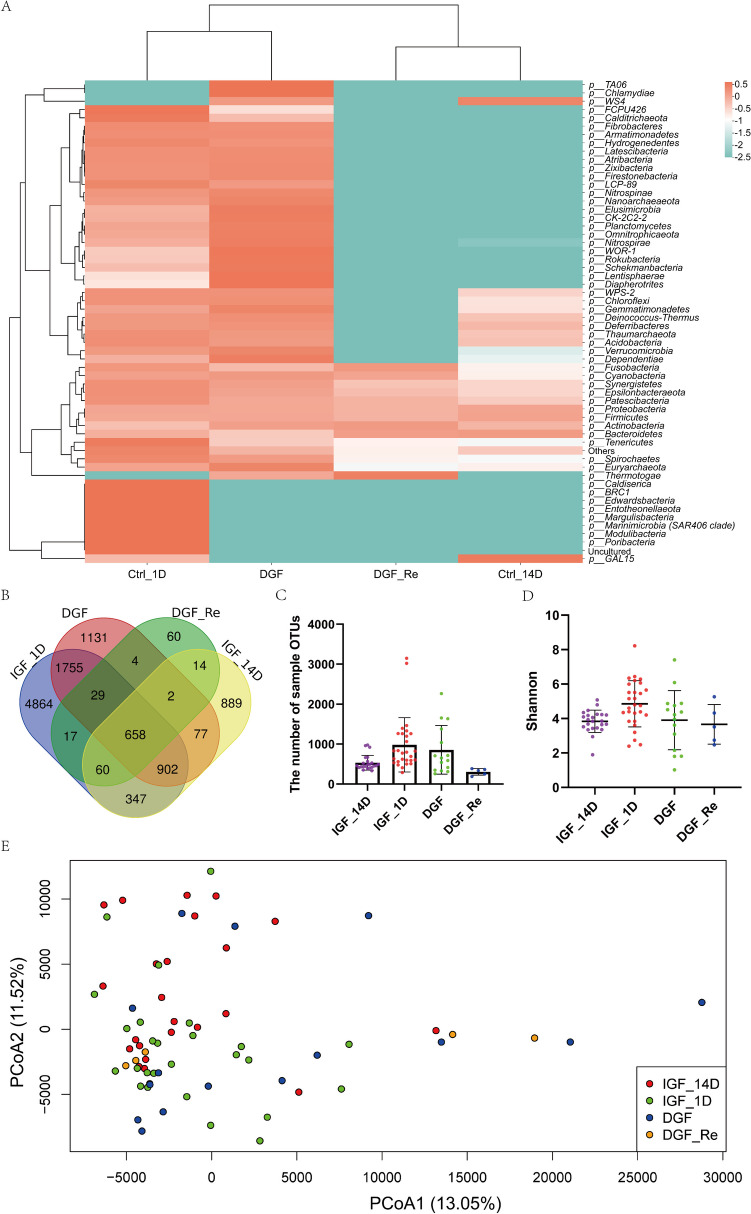
Comparison of the salivary microbiota composition of DGFs and IGFs: **A** species clustering heatmap at phylum level of DGF, DGF_Re, IGF_1D, and IGF_14D groups; **B** Venn graph for the group OTUs of DGF, DGF_Re, IGF_1D, and IGF_14D groups; **C** the number of sample OTUs of DGF, DGF_Re, IGF_1D, and IGF_14D groups; **D** Shannon index of DGF, DGF_Re, IGF_1D, and IGF_14D groups; **E** PCoA graph of DGF, DGF_Re, IGF_1D, and IGF_14D groups. DGF, delayed graft function; IGF, immediate graft function; OTU, operational taxonomic unit; PCoA, principal coordinate analysis

To understand the differences in salivary microbiota composition between DGFs and IGFs with different Scr levels, DGF, DGF_Re, IGF_1D, and IGF_14D groups were compared. The species clustering heatmap at the Phylum level (Fig. 2A) showed that the DGF and IGF_1D groups with similar Scr levels were clustered together, as were the DGF_Re and IGF_14D groups. Nevertheless, quite differences in the relative abundance of taxa were also observed between respective two groups that clustered together, especially *Chlamydiae*, *TAO6*, *Diapherotrites*, *Lentisphaerae*, *Poribacteria*, *Modulibacteria*, and *Margulisbacteria* between the DGF and IGF_1D groups. Furthermore, the number of group OTUs and sample OTUs and the within-group differences decreased following recovery of renal function between group DGF and DGF_Re-like IGFs (Fig. 2 B and C; DGF vs. DGF_Re, *p* = 0.0193; IGF_1D vs. IGF_14D, *p* = 0.0001). The number of sample OTUs of the DGF group was less than that of the IGF group (DGF vs. IGF_1D, *p* = 0.3977; DGF_Re vs. IGF_14D, *p* = 0.0133). As the recovery of renal function, the Shannon index and within-group differences of DGFs also exhibited a downward trend (Fig. 2D; DGF vs. DGF_Re, *p* = 0.7268; IGF_1D vs. IGF_14D, *p* = 0.001). From the ADONIS analysis of beta diversity (Fig. 2E), there was a significant difference between DGF vs. IGF_1D (*p* = 0.003) and DGF_Re vs. IGF_14D (*p* = 0.011), but no significant difference between DGF vs. DGF_Re (*p* = 0.646).

In summary, the microbial composition of the DGF group was significantly different from that of the IGF group and showed less diversity, even when compared at a similar Scr level.

### Prediction of DGF in the early stage based on salivary microbiota

Due to the significant difference in the salivary microbiota composition between the DGF and IGF_1D groups, we aimed to investigate the ability of salivary microbiota to predict DGF in the early-stage post-kidney transplantation.

We utilized LEfSe analysis to identify differential species between the DGF and IGF_1D groups, selecting 33 taxa with LDA values greater than 2 (Supplemental Table S1). We performed univariate binary logistic regression using the logarithmic deformed form (Table 3) and the original value form (Supplemental Table S2) of the relative abundance of these 33 taxa with the grouping information. *Aggregatibacter*, *Corynebacterium*, *Lachnoanaerobaculum*, *Leptotrichia*, and *Selenomonas 3* were significantly correlated with DGF in both analyses. Additionally, we performed a stepwise multivariate binary logistic regression in the original form. Results showed that *Gemella*, *Haemophilus*, and *Lachnoanaerobaculum* played a significant role in this model, and the area under curve (AUC) of the ROC curve was 0.9238 (Supplemental Table S2 and Supplemental Fig. S3E).

**Table 3:** Binary logistic regression and randomForest analysis with the logarithmic form of relative abundance of taxa to classify DGFs and IGFs in 1 day after kidney transplantation

	Univariate analysis		RandomForest analysis	
	OR	*p*	Mean decrease accuracy	Mean decrease Gini
*f__Atopobiaceae*	0.2626	**0.0213**	4.4583	0.6978
*f__P5D1-392*	1.0236	0.6071	0.5947	0.2908
*f__Saccharimonadaceae*	0.2542	**0.0187**	− 0.3105	0.1751
*g__Acinetobacter*	1.0169	0.1600	− 1.7477	0.2544
*g__Aggregatibacter*	0.1446	**0.0058**	0.4477	0.2032
*g__Alloprevotella*	1.0212	0.6099	− 2.3836	0.2379
*g__Anaeroglobus*	0.9815	**0.0104**	− 0.1528	0.2299
*g__Bergeyella*	0.2547	**0.0233**	0.0029	0.3360
*g__Campylobacter*	0.3630	**0.0350**	0.0810	0.3336
*g__Capnocytophaga*	0.1818	**0.0111**	2.1129	0.5552
*g__Comamonas*	0.2397	**0.0120**	− 1.8539	0.1475
*g__Corynebacterium*	0.2597	**0.0134**	3.9397	0.7528
*g__Eikenella*	0.9844	0.2409	0.3516	0.6078
*g__F0058*	0.2964	**0.0134**	0.6685	0.2657
*g__F0332*	0.9874	0.2117	1.7108	0.5514
*g__Gemella*	0.3068	**0.0406**	− 0.6124	0.3888
*g__Gracilibacteria bacterium oral taxon 873*	0.9867	0.0567	− 0.0783	0.2296
*g__Haemophilus*	0.0901	**0.0088**	3.3886	0.8008
*g__Lachnoanaerobaculum*	0.1590	**0.0117**	− 0.4228	0.2315
*g__Lactobacillus*	3.0356	**0.0174**	4.4357	0.5747
*g__Lautropia*	0.3720	**0.0466**	3.7349	0.4471
*g__Leptotrichia*	0.2119	**0.0020**	2.9922	0.7018
*g__Neisseria*	0.1565	**0.0024**	3.6965	0.7978
*g__Parvimonas*	0.2604	0.0550	0.5027	0.2366
*g__Prevotella2*	0.9920	0.5929	− 0.0561	0.5018
*g__Prevotella6*	0.9916	0.3061	− 0.8150	0.1744
*g__Prevotella7*	0.4770	0.0875	− 2.5052	0.2499
*g__Ruminococcaceae UCG-014*	0.9920	0.5926	0.0488	0.3292
*g__Selenomonas*	0.2117	**0.0111**	3.5469	0.9560
*g__Selenomonas3*	0.1058	**0.0033**	5.9961	0.9067
*g__Selenomonas4*	0.9919	0.5897	0.4245	0.5811
*g__Tannerella*	0.3444	**0.0345**	0.9297	0.2746
*g__candidate division SR1 bacterium MGEHA*	0.9851	**0.0335**	− 0.5909	0.2190

*DGF*, delayed graf function; *IGF*, immediate graft function; *OR*, odds ratio

All *p* values less than 0.05 were highlighted in bold

Subsequently, in order to further improve the credibility of the prediction model, we conducted a randomForest analysis. Results showed that the model in logarithmic form (Table 3 and Supplemental Fig. S3A, accuracy = 0.8333, sensitivity = 0.7778, and specificity = 1) was more accurate than the model in original form (Supplemental Table S1 and Supplemental Fig. S2C, accuracy = 0.75, sensitivity = 0.7778, and specificity = 0.6667). Both models had an AUC value of 0.85 (Supplemental Fig. S3B and S3D). Moreover, *Selenomonas 3* played a significant role in differentiating DGF from IGF, whether in logarithmic or original form, in univariate logistic regression analysis, or in randomForest analysis. The relative abundance of *Selenomonas 3* in only one sample in the DGF group was greater than the mean relative abundance of *Selenomonas 3* in the IGF_1D group (Supplemental Fig. S3F).

In conclusion, salivary microbiota was a sensitive and specific predictor of DGF despite similar Scr levels, and *Selenomonas 3* had an important role in the prediction model.

### The correlation of salivary microbiota and renal function in DGF patients

**Fig. 3:**
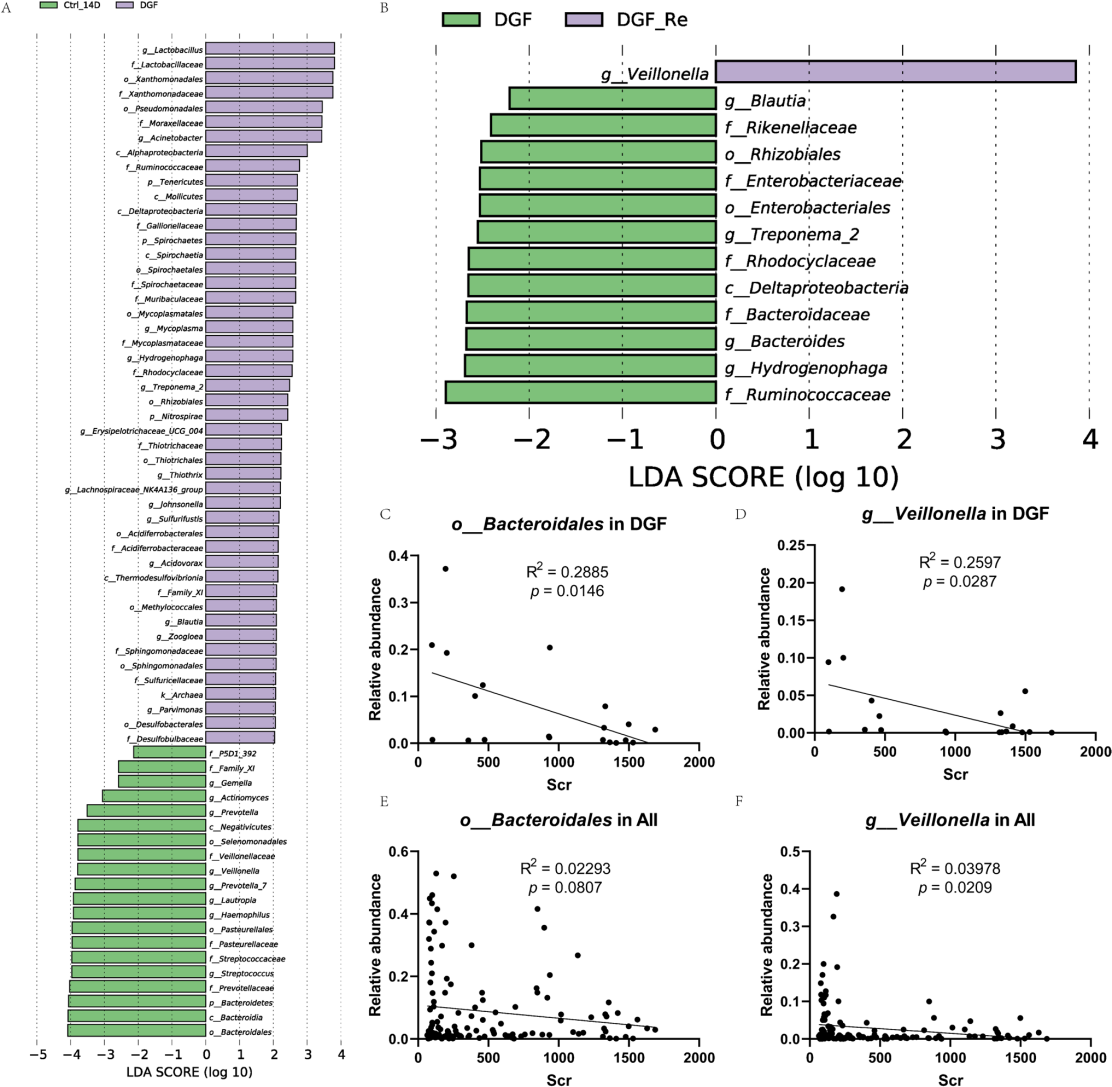
The linear regression between the *Bacteroidales* or *Veillonella* and Scr in patients with DGF and IGF: **A** LEfSe analysis of the salivary microbiota composition between DGF and IGF_14D groups; **B** LEfSe analysis of the salivary microbiota composition between DGF and DGF_Re groups; **C** The scatter diagram of relative abundance of *Bacteroidales* and Scr in DGFs; **D** the scatter diagram of relative abundance of *Veillonella* and Scr in DGFs; **E** the scatter diagram of relative abundance of *Bacteroidales* and Scr in IGFs; **F** the scatter diagram of relative abundance of *Veillonella* and Scr in IGFs. LEfSe, linear discriminant analysis effect size; DGF, delayed graft function; IGF, immediate graft function

To identify potential biomarkers for post-transplant recovery in DGF patients, we selected colonies with LDA values greater than 3 or the highest LDA values from the LEfSe analysis between the DGF and either the DGF_Re or IGF_14D groups for further analysis (Fig. 3 A and B). We identified 13 microbial species and examined their correlation with Scr levels in DGFs and IGFs (Table 4).

**Table 4:** The correlation between the certain taxa and Scr in patients with DGF and IGF

	DGF		IGF	
	Spearman’s *r*	*p*	Spearman’s *r*	*p*
***c__Alphaproteobacteria***	0.3579	0.1213	0.2051	**0.0286**
***f__Ruminococcaceae***	0.3203	0.1686	0.4115	** < 0.0001**
***f__Xanthomonadaceae***	0.0075	0.9749	0.2438	**0.0090**
***g__Acinetobacter***	0.1595	0.5019	0.1520	0.1065
***g__Lactobacillus***	0.0496	0.8354	0.0948	0.3156
*o__Bacteroidales*	− 0.4872	**0.0293**	0.0311	0.7423
*g__Actinomyces*	− 0.3053	0.1906	0.0575	0.5435
*g__Haemophilus*	− 0.2120	0.3695	− 0.1184	0.2097
*g__Lautropia*	− 0.0977	0.6818	− 0.2337	**0.0123**
*g__Prevotella*	− 0.0993	0.6772	0.1172	0.2145
*g__Prevotella_7*	− 0.2286	0.3324	− 0.0497	0.5993
*g__Streptococcus*	− 0.1910	0.4199	− 0.1291	0.1711
*g__Veillonella*	− 0.5474	**0.0125**	− 0.1710	0.0688

The species expressed in bold were those enriched in the DGF group

*DGF*, delayed graf function; *IGF*, immediate graft function

All *p* values less than 0.05 were highlighted in bold

Among the DGFs, only two taxa, *Veillonella* and *Bacteroidales*, were significantly correlated with Scr, prompting us to perform a linear regression analysis to evaluate their relative abundance with Scr levels across different groups (Fig. 3C–H). Our results showed that *Veillonella* had a similar and more pronounced correlation with Scr levels in both DGF and IGF patients, while *Bacteroidales* showed a significant correlation with Scr only in DGF patients.

### Alterations in overall salivary microbiota composition associated with DGF do not appear to persist during long-term follow-up

Further exploration into the longitudinal changes of salivary microbiota in recipients with DGF during long-term follow-up was conducted. A total of 6 recipients previously experiencing DGF (group DGFLT, including three from the original cohort and three additional recruits) and ten IGF recipients (group IGFLT) were enrolled. Their demographic information and laboratory findings are detailed in Table 5. Despite further recovery in renal function as acute kidney injury subsided, DGF patients still exhibited inferior renal function indicators like Scr compared to IGF patients. Additionally, DGF patients in the DGFLT group demonstrated significantly lower hemoglobin concentrations than those in the IGFLT group.

**Table 5:** General characteristics of DGFLTs and IGFLTs

	DGF (*n* = 6)	IGF (*n* = 10)	*p*
Gender, *n* (%)			1.000^#^
Male	5 (83.33%)	9 (90.00%)	
Female	1 (16.67%)	1 (10.00%)	
Age (years), mean ± SD	45.17 ± 7.52	45.30 ± 6.26	0.9809
Immunosuppressive therapy, *n* (%)			0.5170^#^
Tacrolimus + mycophenolic acid + steroids	4 (66.67%)	9 (90.00%)	
Ciclosporin A + mycophenolic acid + steroids	2 (33.33%)	1 (10.00%)	
Immunosuppressant concentration (ng/mL), mean ± SD			
Tacrolimus	7.20 ± 3.38	5.68 ± 1.22	0.7385
Ciclosporin A	NA	NA	
Scr (μmol/L), mean ± SD	139.00 ± 27.91	93.40 ± 20.59	**0.0054**
eGFR (mL/min/1.73 m^2^), mean ± SD	51.69 ± 9.99	83.11 ± 17.22	**0.0005**
BUN (mmol/L), mean ± SD	9.09 ± 1.68	6.18 ± 1.20	**0.0030**
Uric acid (μmol/L), mean ± SD	381.50 ± 65.61	331.2 ± 38.42	0.0993
ALT (U/L), mean ± SD	17.50 ± 3.94	16.50 ± 6.22	0.5100
AST (U/L), mean ± SD	20.50 ± 4.32	20.50 ± 3.50	0.8099
ALB (g/L), mean ± SD	44.10 ± 5.39	45.95 ± 2.76	0.5442
Hb (g/L), mean ± SD	120.20 ± 20.07	141.40 ± 17.65	**0.0335**
PLT (*10^9^/L), mean ± SD	187.70 ± 30.50	212.20 ± 68.68	0.4131

*Scr*, serum creatinine; *eGFR*, estimated glomerular filtration rate; *BUN*, blood urea nitrogen; *ALT*, aminoleucine transferase; *AST*, aspartate aminotransferase; *ALB*, albumin; *Hb*, hemoglobin; *PLT*, platelet

^#^Analyzed with Fisher’s exact test

All *p* values less than 0.05 were highlighted in bold

**Fig. 4:**
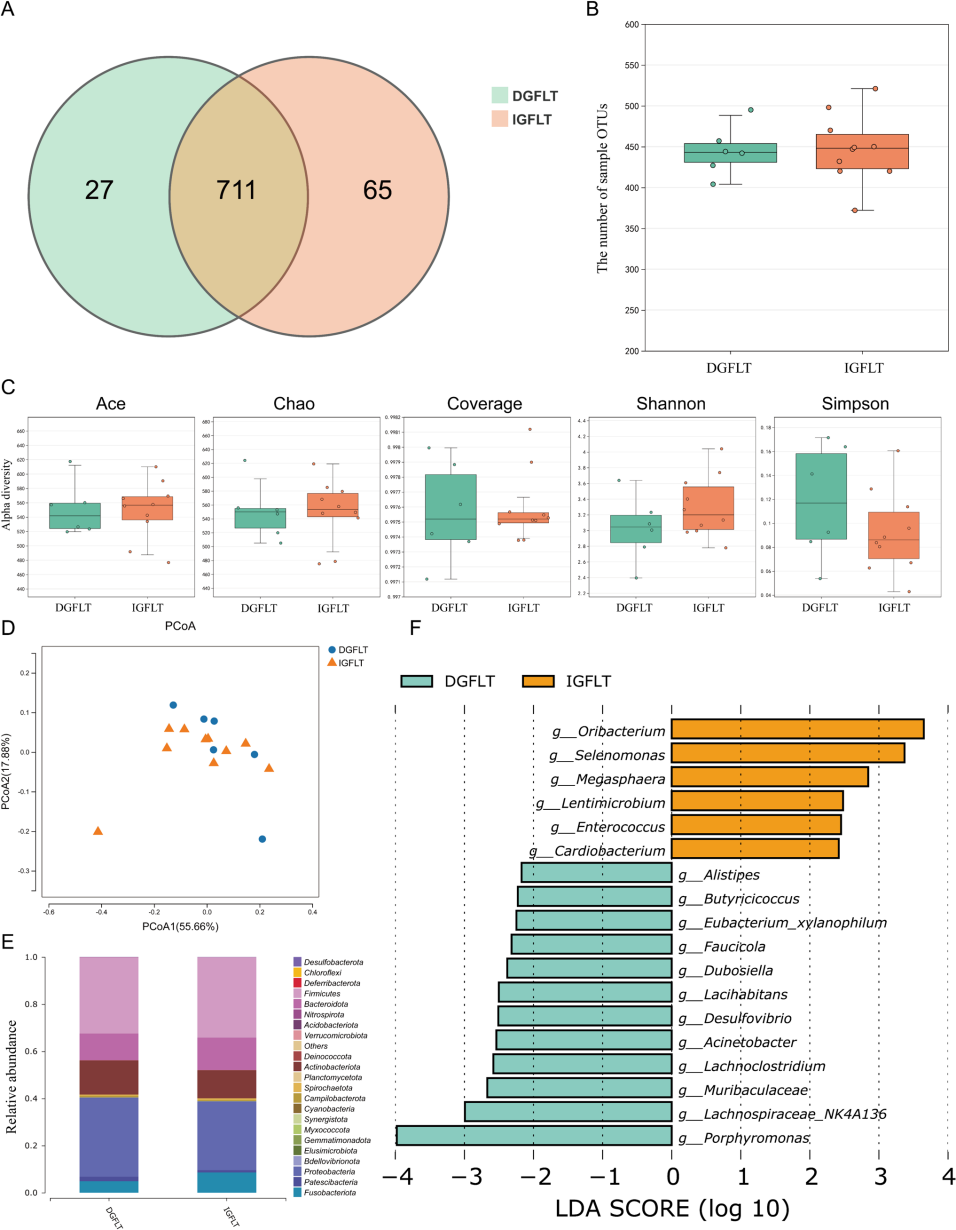
Composition characteristics of salivary microbiota in patients with DGF and IGF after long-term follow-up: **A** Venn graph for the group OTUs of DGFLT and IGFLT groups; **B** the number of sample OTUs of DGFLT and IGFLT groups; **C** Alpha diversity of DGFLT and IGFLT groups; **D** PCoA graph of DGFLT and IGFLT groups; **E** salivary species composition of DGFLT and IGFLT groups at phylum level; **F** LEfSe analysis of the salivary microbiota composition between DGFLT and IGFLT groups. LEfSe, linear discriminant analysis effect size; DGF, delayed graft function; IGF, immediate graft function

The salivary microbiota of both patient groups were compared after long-term follow-up. Results revealed that over 90% of group OTUs were shared between the two groups (Fig. 4A), and there was no significant difference in the number of sample OTUs (Fig. 4B). Five alpha diversity indices, including Ace, Chao, Coverage, Shannon, and Simpson, all indicated no significant differences between the two groups (Fig. 4C). Similar results were also found in beta diversity comparison (Fig. 4D, p = 0.5517). DGFLTs and IGFLTs exhibited similar microbial distribution at the phylum level (Fig. 4E), with only the relative abundance of *Desulfobacterota* showing a difference between the two groups (DGFLT vs. IGFLT, 0.0911% ± 0.0354% vs. 0.0416% ± 0.0119%, *p* = 0.0047). At the Genus level, LEfSe analysis identified 18 differential taxa (Fig. 4F), with the genus *Porphyromonas* significantly enriched in the DGFLT group and the genus *Oribacterium* significantly enriched in the IGFLT group.

## Discussion

In this study, we verified the dynamic change of the salivary microbiota of IGF patients during the perioperative period with a larger sample size. The salivary microbiota of IGF patients significantly differed from that of ESRD patients. The diversity and within-group differences significantly and gradually stabilized following the recovery of renal function, and several taxa were associated with Scr. These results suggested that the salivary microbiota might be the biomarkers for the recovery of renal function as well as the prediction of DGF. Significant diversion was also observed in salivary microbiota between group DGF and ESRD. The comparison of the salivary microbiota between the DGF and IGF groups proved the significant difference between the two groups, even though at a similar Scr level. The prediction model of DGF using the salivary microbiota detected at 24 h post-transplantation was developed by the randomForest analysis and showed good performance. In addition, *Bacteroidales* and *Veillonella* were found to be significantly associated with perioperative Scr in patients with DGF.

In agreement with the previous study (Xiang et al. 2023), the salivary microbiota composition during the perioperative period of IGF patients differed significantly from that of ESRD patients. The salivary microbiota of IGF patients gradually stabilized, manifesting as the diversity minimized and the intra-group variation declined following the recovery of renal function. A broader range of taxa of IGF patients was explored for correlation with Scr. Spearman analysis showed that *Patescibacteria* (*p* < 0.0001) and *Leptotrichiaceae* (*p* = 0.0001) were positively correlated with Scr after kidney transplantation, consistent with the previous research. Multiple linear regression analysis was then performed with Scr, and the meaningful taxa were screened out according to the absolute values of Spearman’s *r* > 0.4 and *p* < 0.0001 from Spearman analysis. We found that *Dialister*, *F0058*, *Parvimonas*, and *Selenomonas* were significantly and independently associated with Scr. They have been found to be correlated with kidney disease in several studies. *Dialister* is a genus of *Bacillota* bacteria classified within the class *Negativicutes*. A large human cohort study identified and validated a significant association between *Dialister* and chronic kidney disease, and *Dialister* has the potential to be a diagnostic tool for chronic kidney disease and could be used to reflect disease progression and circulating nephrotoxin levels as a gut taxa (Wu et al. 2020). Meanwhile, *Dialister* was also found to be enriched in the salivary taxa of patients with Sjögren’s syndrome (Sharma et al. 2020). In our clinical observations, salivary hypersecretion was also found in patients in the early post-transplant period after kidney transplantation. Therefore, it was not surprising that *Dialister* is significantly and independently associated with short-term prognosis in patients after kidney transplantation. *Parvimonas*, a gram-positive anaerobic coccus, is commonly found in dental plaque of chronic periodontitis patients. *Parvimonas* was also closely associated with the development of periodontitis in hemodialysis and solid organ transplant patients (Schmalz et al. 2016, 2017, 2018).

Similarly, to investigate the role of salivary microbiota in the recovery of DGF, LEfSe analysis was conducted to screen taxa, and the Spearman test analyzed the correlation between each taxon and Scr. Results revealed that *Bacteroidales* and *Veillonella* were significantly associated with Scr level in DGF. Moreover, *Veillonella* also showed a weaker correlation with Scr in IGF (Spearman’s *r* =  − 0.171, *p* = 0.0688). The role of *Bacteroidales* and *Veillonella* in the digestive tract in the process of kidney injury has been investigated. *Bacteroidales* is an order of bacteria. Notably, it includes the genera *Prevotella* and *Bacteroides*, which are commonly found in the human gut microbiota (Wu et al. 2016). *Bacteroidales* have been strongly and paradoxically associated with kidney disease in the last decade. Chen et al. (2018) discovered the protective effects of polysaccharides from mulberry fruit on diabetic db/db mice, accompanied by an enrichment of *Bacteroidales* in the intestinal microbiota. You-gui pill is a classic Chinese medicine comprising ten traditional Chinese medicines like Radix Rehmanniae Praeparata. It protects against renal dysfunction by regulating the gut microbiota and its metabolic pathways, which includes an increase in the relative abundance of *uncultured_bacterium_f_Bacteroidales_S24-7_group* (Chen et al. 2019). Among antineutrophil cytoplasmic antibody-associated vasculitis with kidney injury patients, *unclassified_o_Bacteroidales* was correlated negatively with serum creatinine which was consistent with our study (Yu et al. 2022). Contrary to the above research, for the modified chronic renal failure model rats, the administration of total flavones of *Abelmoschus manihot* and the drug febuxostat improved renal injury and remodeled gut microbiota dysbiosis, including decreased *Bacteroidales* and *Lactobacillales* and increased *Erysipelotrichales* (Tu et al. 2020). Intestinal microbiome changes in lupus-like mice showed reduced diversity, with increased *Bacteroidetes* and decreased *Firmicutes* abundance (He et al. 2020). Fecal microbiota transplant worsened the kidney injury of SS/CRL mice responding to a high-salt diet, along with increasing relative abundance of *Bacteroidales_1* (Abais-Battad et al. 2021). *Bacteroidales* was closely associated with kidney injury, but the role they played in DGF still needed to be further explored. *Veillonella* was less frequently studied in kidney disease. *Veillonella*, a non-fermentative, strictly anaerobic, gram-negative coccus is present in the human gastrointestinal tract, mouth, and vaginal microbiota. In chronic kidney disease patients, Hu et al. (2018) observed a significant depletion of *Veillonella* in the oral microbiota of saliva and anterior mandibular lingual sample types. In summary, these two taxa were associated with kidney damage and were significantly correlated with Scr in patients with DGF. The distribution of them may be predictive of a perioperative and more distant prognosis in patients with DGF, which would require a larger and more comprehensive sample size to prove.

Currently, only one study directly discusses the relationship between salivary microbiota and renal function. In a case–control study recruiting IgA nephropathy patients and healthy individuals, two OTUs were found to be significantly negatively correlated with Scr, but the study did not annotate these OTUs (Luan et al. 2019). Nevertheless, comparing with other studies, which explore the link between salivary microbiota and kidney diseases, reveals some interesting parallels. Patients with diabetic nephropathy show an enrichment of *Selenomonas* in saliva compared to those without diabetic nephropathy but with chronic periodontitis (Zhang et al. 2022). Similarly, *Selenomonas* positively correlated with Scr in IGFs. Furthermore, *Veillonella*, which is depleted in individuals with IgA nephropathy compared to healthy controls (Piccolo et al. 2015; Luan et al. 2019), was negatively associated with Scr in patients with DGF. Hence, we speculate a potential association between *Selenomonas* and *Veillonella*, and Scr, suggesting promising biomarkers for discerning renal function impairment.

There was a significant difference in the overall composition of salivary microbiota between DGF and IGF patients, as demonstrated by ADONIS analysis of beta diversity. Nonetheless, the species clustering heatmap, the number of group or sample OTUs, and the Shannon index also showed significant differences in salivary microbiota between DGF and IGF patients even though at similar levels of Scr, suggesting that DGF-related acute kidney injury, secondary systemic inflammation, etc. significantly altered the distribution of salivary microbiota in patients. Although there was no study indicating the association between the change of salivary microbiota and acute kidney injury, research has shown the changes in gut microbiota caused by acute kidney injury. The study identified increased *Enterobacteriacea* and decreased *Lactobacill*i and *Ruminococacceae* as key indicators of dysbiosis induced by ischemia/reperfusion injury in mice. These changes were linked to reduced levels of short-chain fatty acids, intestinal inflammation, and leaky gut (Yang et al. 2020). Andrianova et al. (2020) identified correlations between bacterial abundance and metabolite levels in rats with ischemic kidney injury. *Rothia* abundance correlated positively with creatinine levels, while *Staphylococcus* abundance correlated positively with urea levels. The acute and subchronic toxicities of desaminotyrosine, which led to acute kidney injury, concentration-dependently affected the diversity of intestinal microbiota (Li et al. 2021). Salivary microbiota had also been shown to be associated with many systemic inflammatory conditions. Not only colitis (Qian et al. 2022) but also Alzheimer’s disease (Lu et al. 2022), depression (Kelly et al. 2016), psoriasis (Belstrøm et al. 2020), and tumors (Tuominen and Rautava 2021) were closely related to salivary microbiota. Therefore, the alternation of salivary microbiota between DGF and IGF patients may be associated with acute kidney injury and secondary systemic inflammation.

Additionally, when the renal function of DGF patients gradually recovered, the same trend of salivary microbiota was observed in DGF patients as IGF, such as the decrease in the number of group OTUs and sample OTUs, the decline in the Shannon index, and the minimization in the intra-group differences of several indices. These results suggested that the recovery of renal function may bring significant and similar variations in the salivary microbiota composition of both IGF and DGF. The relationship between digestive tract microbiota and kidney function has been extensively studied, and the two are mutually causal. As kidney function deteriorated and chronic kidney disease progressed, during which it was accompanied by dysbiosis in the digestive tract microbiota. Different changes could be found in the gut microbiota of patients with early (Sato et al. 2021), progressive (Li et al. 2019; Kim et al. 2020), and end-stage (Wang et al. 2020) chronic kidney disease that the variations of alpha diversity, beta diversity, feature taxa, and number of OTUs differed in distinct stages. In turn, these changes in gut microbiota could often drive the progression of chronic kidney disease. Wang et al. (2020) found that *Eggerthella lenta* and *Fusobacterium nucleatum* were enriched in the gut of patients with ESRD. These taxa increased the production of uraemic toxins and promoted renal disease development in a chronic kidney disease rat model. *Faecalibacterium* was significantly depleted in patients with chronic kidney disease, which would reduce renal dysfunction through the G protein-coupled receptor-43 in chronic kidney disease mice (Li et al. 2022). The damage of these taxa to the kidney was related to the production of their uremic toxins (Gryp et al. 2020). Therefore, we were not surprised that the recovery of renal function significantly affected the composition of salivary microbiota of both DGF and IGF.

There were numerous diagnostic criteria for DGF, including the need for dialysis within 1 week after transplantation (Ojo et al. 1997), a decrease in serum creatinine of less than 10% for three consecutive days within 1 week after transplantation (Boom et al. 2001), and Scr greater than 400 µmol/L 7 days after transplantation (Mallon et al. 2013). The diagnosis of DGF requires at least 72 h after kidney transplantation. However, earlier management and intervention were important for the prognosis of patients with DGF. Therefore, there is a need for biomarkers that could predict the occurrence of DGF in the early stage. Given the significant difference in salivary microbiota composition between DGF and IGF, we analyzed the salivary microbiota composition of the DGF and IGF_1D groups to evaluate the ability of salivary microbiota to distinguish DGF from IGF at 1 day after surgery. We performed randomForest analysis using the relative abundance of the screened colonies and found that the models constructed using the logarithmic form demonstrated better discriminatory ability for the DGF (accuracy = 0.8333, sensitivity = 0.7778, specificity = 1, and AUC = 0.85). Therefore, we may be able to determine the prognosis of patients and provide appropriate treatment 24 h after kidney transplantation which help DGF patients achieve a better recovery. In addition, we found that *Selenomonas*, a species enriching in IGF_1D group, played an important role in every classification model. *Selenomonas* has not been studied in the field of kidney disease-related, but it has been found to be inducting inflammation in anatomical sites such as the oral cavity, digestive tract, respiratory tract, and female genital tract. *Selenomonas* is an anaerobic, gram-negative motile bacterium usually found in the oral cavity of humans or the rumen of herbivores (Pomeroy et al. 1987). *Selenomonas* was found to be relevant to periodontal disease as early as the twentieth century (Tanner et al. 1989). Hawkes et al. (2023) discovered that *Selenomonas sputigena* adhered to gingival keratinocytes, prompting the expression and secretion of inflammatory cytokines and chemokines linked to leukocyte recruitment. These cytokines from *S. sputigena*–infected keratinocytes stimulated monocyte and neutrophil chemotaxis. *Porphyromonas* was another major cause of periodontitis, and *Selenomonas* was always counted in conjunction with *Porphyromonas* (Gonçalves et al. 2012). *Selenomonas* was also associated with all-age dental caries disease (Preza et al. 2008; Gross et al. 2010; Ortiz et al. 2019). *Selenomonas* was associated with the development of intra-oral halitosis in both children and adult oral microbiota (Ren et al. 2016; Seerangaiyan et al. 2017), which may explain the bad breath symptoms in patients with DGF and even in patients with chronic kidney disease (Santaella et al. 2021). An elevated abundance of digestive *Selenomonas* was also associated with numerous other systemic diseases, such as bacteremia (McCarthy and Carlson 1981), various digestive tract tumors (Li et al. 2020; Zhang et al. 2021; Zeng et al. 2022), gynecological and pregnancy diseases (Tuominen et al. 2018; Xu et al. 2020; Liu et al. 2021), asthma (Kim et al. 2022), and tuberculosis (Hu et al. 2020), suggesting that *Selenomonas* might be a pro-inflammatory taxon. Interestingly, *Selenomonas* not only played an important role in the early-stage prediction of DGF with lower abundance but also was independently, significantly, and positively associated with Scr of IGF but not DGF. The enrichment of *Selenomonas* in saliva early after kidney transplantation appeared to facilitate the repair of kidney injury, and the relative abundance of it was negatively regulated with the recovery of kidney function. However, the relationship between *Selenomonas* and DGF-related inflammation still needs further evidence.

Several studies have explored the variation of salivary microbiota in patients with kidney diseases. As one kind of kidney disease, the composition of salivary microbiota in DGF patients may differ markedly from that of other kidney disease patients. In our study, DGFs exhibit lower alpha diversity compared to ESRDs, suggesting reduced richness and evenness of microbiota composition, suggesting the more severe microbial dysbiosis. Beta diversity further confirms the significant structural differences in microbiota. Additionally, several differential microbial taxa were found between the two groups. Akin to healthy individuals, DGFs exhibited a lower abundance of *Neisseria* compared to ESRDs (Khasnobish et al. 2021). Conversely, the relative abundance of *Prevotella* (Luan et al. 2019; Liu et al. 2022) and *Gemella* (Piccolo et al. 2015; Zhang et al. 2022), which is downregulated in ESRDs compared to healthy individuals, undergoes further reduction in DGFs. Moreover, when compared to relatively healthy individuals under the similar immunological background, patients with other kidney diseases do not exhibit changes in alpha diversity (Piccolo et al. 2015; Luan et al. 2019; Liu et al. 2022; Zhang et al. 2022) but show significant alterations in beta diversity (Duan et al. 2020; Khasnobish et al. 2021; Liu et al. 2022; Luan et al. 2022), and the trend was similarly observed in DGFs. Interestingly, *Selenomonas*, enriched in diabetic nephropathy patients, undergoes depletion in DGFs, and the opposite is true for *Gemella* (Zhang et al. 2022). In summary, while there are some similarities in the salivary microbiota composition between DGF patients and those with other kidney diseases, numerous findings suggest that the microbial changes in DGF patients are distinctive. Our conclusions offer potential for clinical translation.

The comparison in our cohort after long-term follow-up further elucidated the association between DGF and salivary microbiota characteristics. Results indicated no significant differences in the number of sample OTUs, alpha diversity, beta diversity, and microbial community structure at the phylum level between DGF and IGF patients when DGF patients reached renal function similar to IGF levels. This suggests that acute kidney injury experienced by DGF recipients perioperatively may not have a lasting and profound impact on salivary microbiota features as renal function gradually recovers. Interestingly, some alpha diversity parameters showed a trend of difference, and ADONIS analysis based on unweighted unifrac distances suggested significant differences between the two groups (results not specified, *p* = 0.0167). Additionally, LEfSe analysis identified multiple differential taxa at the genus level. These differences may stem from the persisting differences in renal function between the two groups, supporting the close association between salivary microbiota and renal function post-kidney transplantation and bolstering confidence in further exploration of the relationship between salivary microbiota and outcomes in DGF recipients.

The limitation of our study lies in the small sample size, potentially affecting the reliability of salivary microbiota in predicting DGF. In other cohorts, DGF incidence in KT from donation after circulatory death is 20% (Savoye et al. 2021), likely to be lower from donation after brain death (Rijkse et al. 2021). During our recruitment period, DGF incidence was 11.36%, which will further decrease with a prolonged time, given that nearly all KT recipients at our center receive donations after brain death. Therefore, accumulating a sufficient sample size within a short time frame poses a challenge. The characteristics reported in individual cases may not necessarily generalize to the wider population. Hence, the expanding sample size and external validation are priorities for our future research, along with investigating the role of differential taxa in animal models.

## Supplementary Information

Below is the link to the electronic supplementary material.

Supplementary file1 (PDF 486 KB)

Supplementary file2 (XLSX 17 KB)

## Data Availability

16S rRNA sequencing data are deposited under SRA PRJNA904953 and PRJNA963071.
