# Supplementary material for: Prediction of delayed graft function by early salivary microbiota following kidney transplantation
Source: Appl Microbiol Biotechnol. 2024 Jul 1;108:402. doi: 10.1007/s00253-024-13236-w (PMC11217047; doi:10.1007/s00253-024-13236-w)
Supplement: Supplementary file 1 [file 253_2024_13236_MOESM1_ESM.pdf]

Supplemental Material

Journal name:

Applied Microbiology and Biotechnology

Manuscript Title:

Prediction of Delayed Graft Function by Early Salivary Microbiota Following Kidney Transplantation

The name(s) of the author(s):

Xuyu Xiang1,2,†, Bo Peng1,2,†, Kai Liu1,2, Tianyin Wang1,2, Peng Ding1,2, Yi Zhu1,2, Ke Cheng1,2 and Yingzi Ming1,2,*

The affiliation(s) and address(es) of the author(s):

1 The Transplantation Center of the Third Xiangya Hospital, Central South University, Changsha 410013, China

2 Engineering and Technology Research Center for Transplantation Medicine of National Health

The e-mail address, telephone and fax numbers of the corresponding author:

Yingzi Ming,

mingyz_china@csu.edu.cn

+86 133 0731 8463

No fax numbers

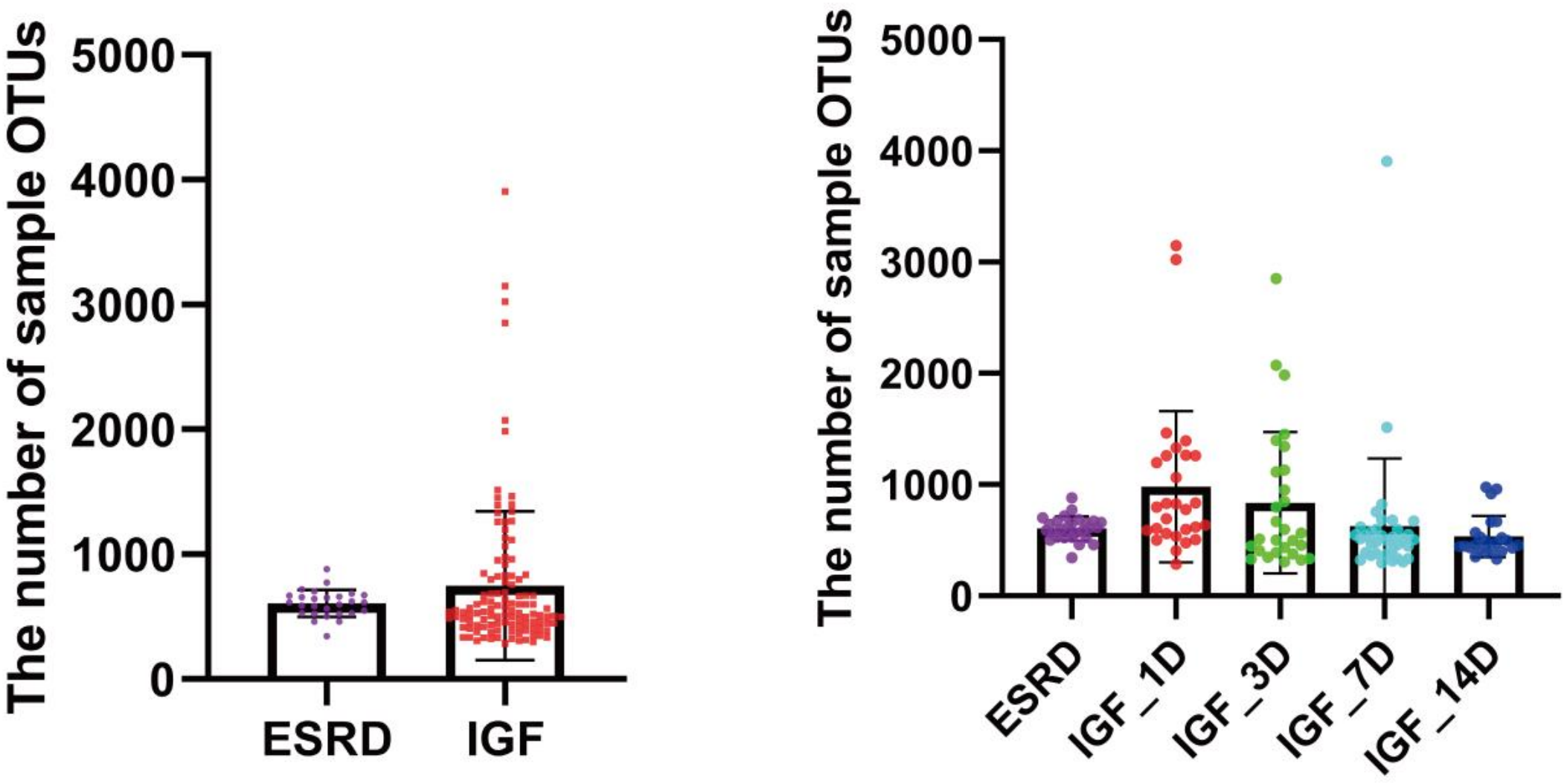

Supplemental Fig. S1. The number of sample OTUs of ESRD, IGF, IGF_1D, IGF_3D, IGF_7D and IGF_14D groups: (A) The number of sample OTUs of ESRD and IGF groups; (B) The number of sample OTUs of ESRD, IGF_1D, IGD_3D, IGF_7D and IGF_14D groups.

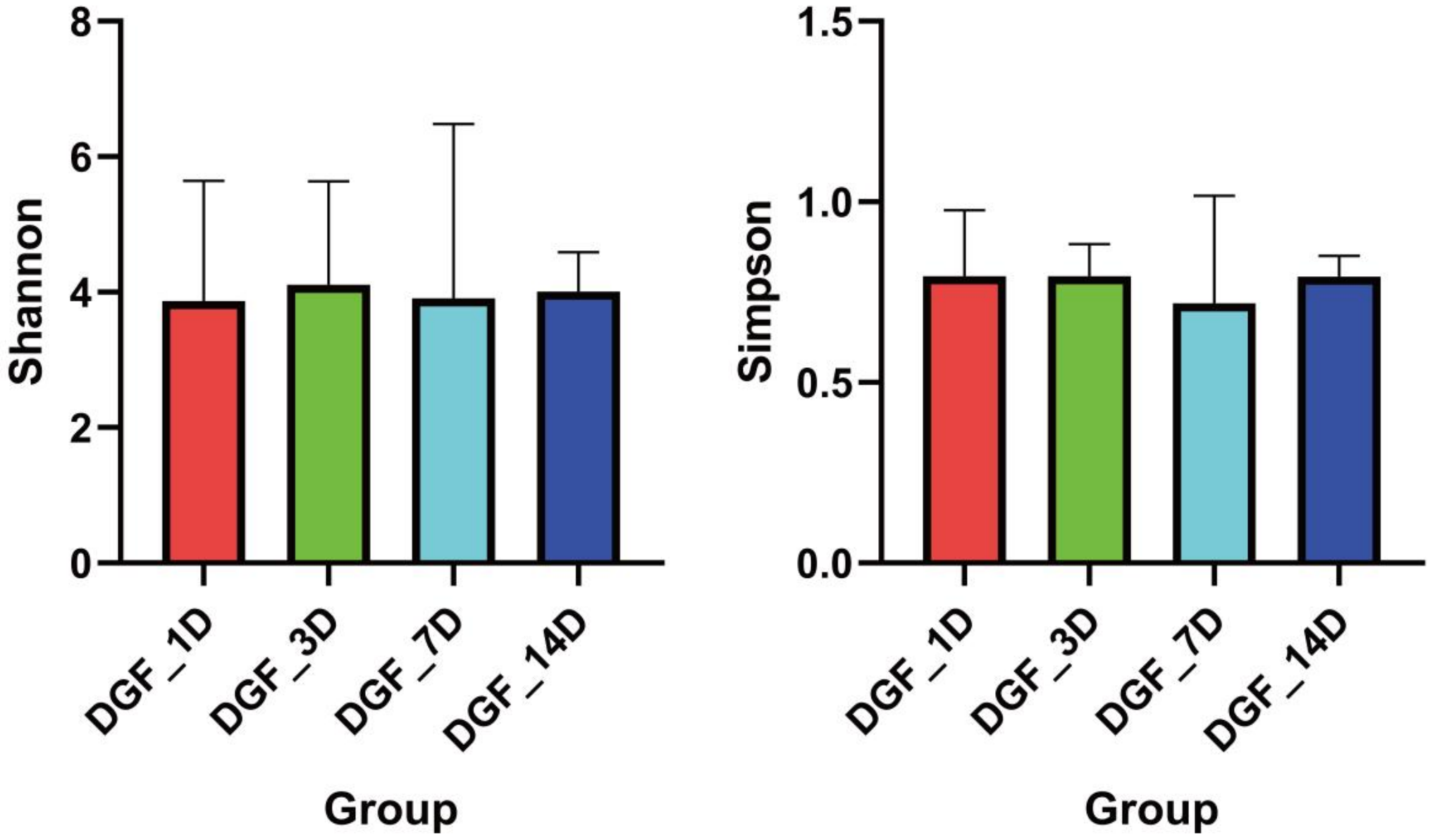

Supplemental Fig. S2. Shannon and Simpson index of DGF_1D, DGF_3D, DGF_7D and DGF_14D: (A) Shannon index of DGF_1D, DGF_3D, DGF_7D and DGF_14D; (B) Simpson index of DGF_1D, DGF_3D, DGF_7D and DGF_14D.

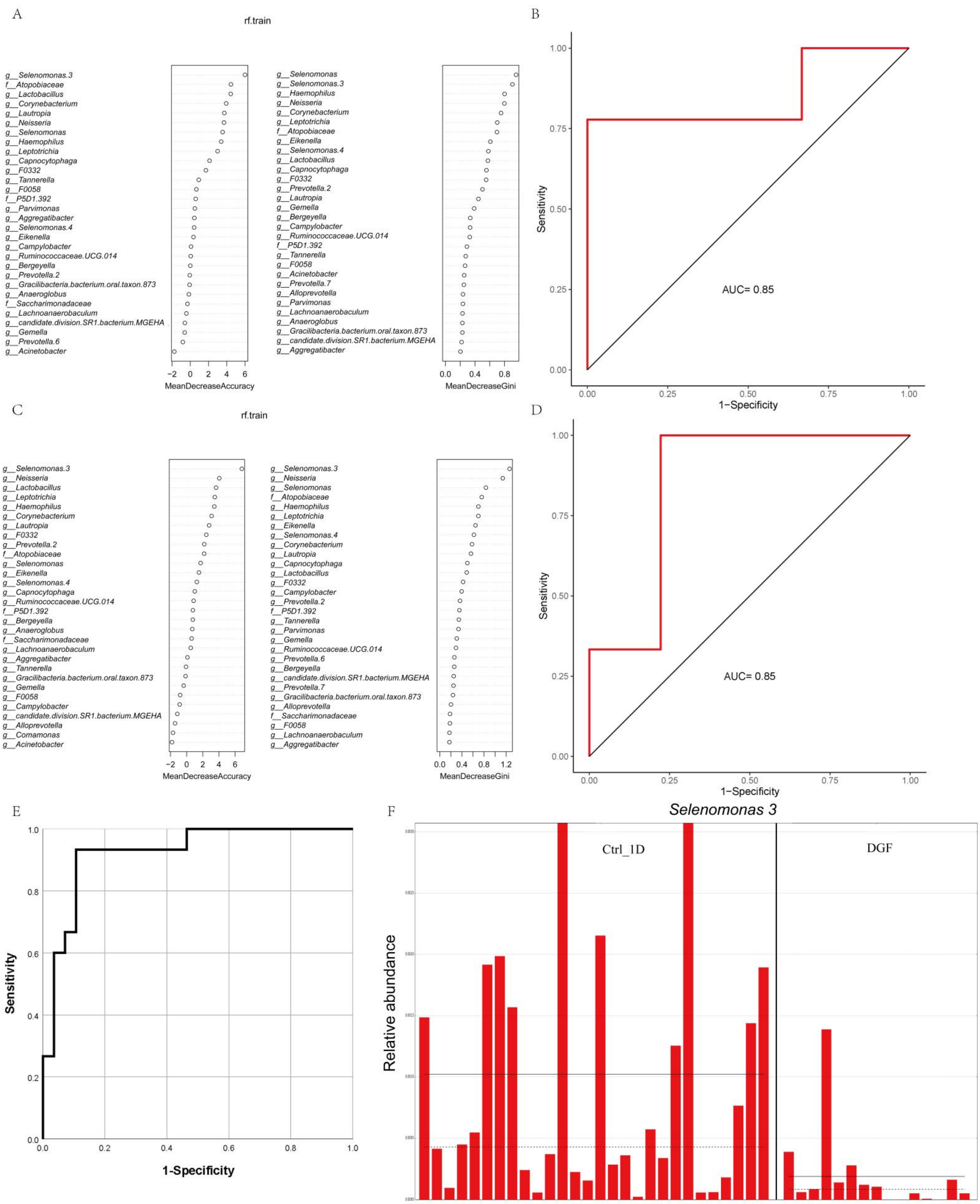

Supplemental Fig. S3. The model evaluation: (A) The mean decrease accuracy and mean decrease gini of taxa in randomForest model in logarithmic form; (B) The ROC curve of randomForest model in logarithmic form; (C) The mean decrease accuracy and mean decrease gini of taxa in randomForest model in original value form; (D) The ROC curve of randomForest model in original value form; (E) The ROC curve of multivariate binary logistic regression model in original value form; (F) The relative

abundance of *Selenomonas 3* in each samples.
